# Efficacy of fermented grain using *Bacillus coagulans* in reducing visceral fat among people with obesity: a randomized controlled trial

**DOI:** 10.3389/fnut.2023.1148512

**Published:** 2023-04-17

**Authors:** Eunbyul Cho, Ju Young Kim, Belong Cho, Joong Su Lee, Yeo Cho Yoon, Yong Chul Shin, Hyerim Kim, Siye Gil, Sohye Kim

**Affiliations:** ^1^Department of Family Medicine, Seoul National University Bundang Hospital, Seongnam-si, Republic of Korea; ^2^Department of Family Medicine, Seoul National University College of Medicine, Seoul, Republic of Korea; ^3^Amicogen, Inc., Jinju-si, Republic of Korea; ^4^Nutrition Care Services, Seoul National University Bundang Hospital, Seongnam-si, Republic of Korea

**Keywords:** visceral fat, anti-obesity, fermented grain, *Bacillus coagulans*, weight management

## Abstract

**Background:**

Obesity is a socioeconomic problem, and visceral obesity, in particular, is related to cardiovascular diseases or metabolic syndrome. Fermented grains and various microorganisms are known to help with anti-obesity effects and weight management. Studies on the relationship between *Bacillus coagulans* and anti-obesity effects are not well known, and studies on the application of fermented grains and microorganisms to the human body are also insufficient.

**Objectives:**

This study aimed to evaluate the efficacy of Curezyme–LAC, an ingredient mixed with six-grain types fermented by *B. coagulans*, in reducing fat mass in adults with obesity.

**Methods:**

In this randomized double-blinded placebo-controlled study, 100 participants [aged 40–65 years; body mass index (BMI) ≥ 25 to ≤ 33 kg/m^2^) were randomly allocated to two groups: 4 g/day Curezyme–LAC administered as a granulated powder or placebo (steamed grain powder mixture).

**Results:**

After 12 weeks, visceral adipose tissue decreased significantly in the Curezyme–LAC group compared with that in the placebo group (mean ± standard error, SE of −9.3 cm^2^ ± 5.1) vs. (6.8 cm^2^ ± 3.4; *p* = 0.008). Compared to the placebo group, the Curezyme–LAC group also showed significant reductions in total fat mass (−0.43 ± 0.24 kg vs. 0.31 ± 0.19 kg, *p* = 0.011), body weight (−0.4 ± 0.3 kg vs. 0.3 ± 0.2 kg, *p* = 0.021), BMI (−0.14 ± 0.12 vs. 0.10 ± 0.07, *p* = 0.028), and waist circumference (−0.6 ± 0.2 cm vs. −0.1 ± 0.2 cm, *p* = 0.018) without a change in dietary intake and physical activity.

**Conclusion:**

Curezyme–LAC supplementation for 12 weeks may benefit individuals with obesity by reducing visceral fat mass.

## 1. Introduction

Obesity has become an important health, social, and economic issue with a rapidly increasing global prevalence—nearly a third of the world’s population is currently classified as overweight or obese (1), and almost half of the world’s adult population will be overweight or obese by 2030 (2). The increase in medical costs due to an increase in the obese population is a socioeconomic problem worldwide, including in South Korea. The aggregate medical costs due to obesity more than doubled over the 16-year period from 2001 to 2016. In 2016, medical expenditure directly attributable to obesity in the United States was $260.0 billion (3). Obesity is a major risk factor for the development of cardiovascular diseases (4); cancer (5); and clinical conditions such as metabolic syndrome, early atherosclerosis, hypertension, diabetes, and dyslipidemia (6). Most importantly, an increase in visceral fat area is related to many metabolic abnormalities, including impaired glucose tolerance, insulin resistance, and increased levels of very low-density lipoprotein triglycerides (VLDL-TG), which are associated with abdominal obesity (7). Visceral fat functions as an endocrine organ with adipocytes secreting various adipocytokines (8), whose levels are increased in obesity-related diseases, such as type 2 diabetes mellitus (T2DM), metabolic syndrome, and cardiovascular diseases (9, 10). Therefore, visceral fat reduction has become an important goal in managing and treating obesity (11).

There have been several studies on various foods, nutrition or ingredients, and microorganisms that can reduce body mass index (BMI) and body fat mass, especially visceral fat (12, 13). Particularly, fermented grains are known to have numerous benefits, including anti-obesity effects (14, 15). Fermented grains contain ordinary nutrients, micronutrients, and biologically active components, including peptides, phenolic compounds, phytosterols, β-glucan, and organic acids (16, 17). During fermentation, lactic acid bacteria produce organic acids, mostly lactic and acetic acids, from raw materials (18). Microbial enzymes, such as amylases, maltases, proteases, cellulases, and phytases, are activated by lowering the pH level, and the breakdown of cereal cell walls is initiated (19). Various health-promoting constituents are released due to cell disruption, and the components generated by fermentation have been reported to possess inhibitory effects against obesity, T2DM, cancer, and cardiovascular disease (20, 21).

Curezyme–LAC is a mixture of six grains processed by fermentation, derived from the metabolism of *Bacillus coagulans*, a lactic acid-producing bacterium. *B. coagulans* is “Generally Recognized As Safe” by the US Food and Drug Administration (22). *B. coagulans* strains have been used in various food products owing to their beneficial food processing and health-promoting effects (23). These strains are spore-forming bacteria that are resistant to high temperatures and acidity (24). *B. coagulans* can survive in gastrointestinal tract conditions better than other probiotic microorganisms (25).

Previously, we investigated the effect of Curezyme–LAC in high-fat diet-induced obese mice and found that it significantly reduced body weight, fat mass, plasma lipid content, and fasting blood glucose levels in a dose-dependent manner. Furthermore, the concentrations of inflammatory cytokines secreted from adipocytes remarkably decrease and improve glucose tolerance (14). This study aimed to investigate the effect of Curezyme–LAC intake on body fat reduction in participants with obesity, as in previous animal studies.

## 2. Materials and methods

### 2.1. Participants and study materials

**FIGURE 1:**
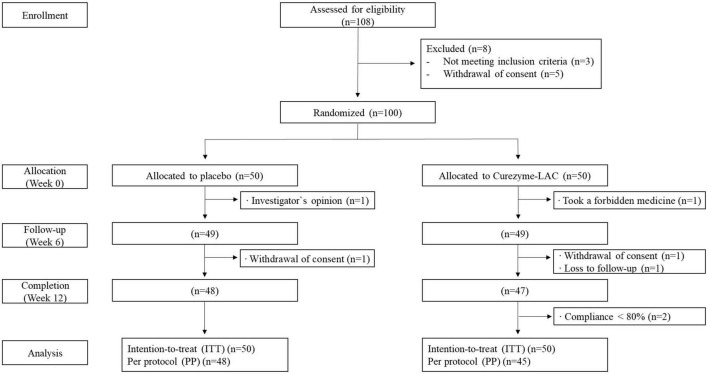
CONSORT diagram for flow of subjects through the study.

Women and men with BMI ≥25 to ≤33 kg/m^2^ aged 40–65 years (*n* = 108) who volunteered and met specified inclusion and exclusion criteria were recruited for this study (Supplementary Table 1) through posters and advertisements. In Western populations, adult obesity is defined as having a BMI of ≥30 kg/m^2^, while being overweight is defined as having a BMI between 25 and 29.9 kg/m^2^ (26). In Asians, adult obesity is defined as BMI ≥25 kg/m^2^, which is equivalent to being overweight by the international definition. This definition for adult Asians was suggested by the WHO in 2000 (27). Furthermore, many studies from Asia still adopt the lower BMI cutoff as detailed in the 2000 proposal (28). For this reason, in this study, we defined obesity as BMI ≥25 kg/m^2^, and this study was conducted on participants with obesity. After being informed clearly and precisely of the study’s objective, protocol, and predictable risks involved in the trial, 108 participants signed a written informed consent form. Eight individuals withdrew consent, and the remaining 100 commenced the study. Seven participants were lost to follow-up or dropped out of the study due to the investigator‘s opinion, taking a forbidden medicine, or withdrawing consent, and 93 participants completed the study (Figure 1). The initial 100 participants were allocated in a 1:1 ratio to receive either a placebo (*n* = 50) or Curezyme–LAC (*n* = 50) using a computer-generated random number list. The participants received a daily dose of 4 g of Curezyme–LAC or placebo and were instructed to take two sachets of 2 g twice daily, 30 min before meals with water over 12 weeks.

Curezyme–LAC was kindly provided by Amicogen Inc. (Jinju, Korea). To prepare the placebo, six types of grains (Sanlim global Corp., Yongin, Korea)—soybean, barley, job’s tear, corn, wheat, and brown rice—were steamed with water at 120°C and dried at 60°C. To prepare the Curezyme–LAC sample, the same six types of grains used for the placebo were fermented separately in the solid and liquid state with *B. coagulans* KCTC13284BP in an aseptic ventilation facility at 35°C and dried at 60°C. A 3 g test powder contained 2,000 mg of Curezyme–LAC (fermented mixed grains) or steamed mixed grains powder along with vehicle material (Supplementary Table 2). The placebo had the same formulation with identical flavor and taste as the test material, except that Curezyme–LAC was replaced by steamed mixed grain powder.

The dosage of Curezyme–LAC applied to the participants had been determined from a previous animal study using high-fat-diet-induced obese C57BL/6J mice fed with 732 mg/kg of Curezyme–LAC. This study showed a significantly decreased body weight and fat mass with simultaneous reductions in plasma lipid content without any adverse events (18). Thus, 4,000 mg of Curezyme–LAC was considered appropriate for humans with an average body weight of 60 kg according to the guidance for estimating the maximum safe starting dose in initial clinical trials for therapeutics in adult healthy volunteers (19).

### 2.2. Study design and intervention

This 12-week double-blinded randomized parallel placebo-controlled trial was performed at two medical centers in South Korea: Seoul National University Hospital and Seoul National University Bundang Hospital. Written informed consent was obtained from all participants for inclusion in the study before participation. All research procedures conducted in this trial were in accordance with a protocol approved by the Institutional Review Board (IRB) of Seoul National University Hospital as well as by Seoul National University Bundang Hospital and adhered to the Declaration of Helsinki (IRB No. H-2002-098-1103). This trial was registered in the Clinical Research Information Service with the identification number KCT0005095.

All participants were instructed to maintain their usual dietary patterns and physical activity during the study period. Dietary intake was assessed using a standardized food frequency questionnaire and a 3-day dietary record to obtain total daily energy with macronutrient intake every 6 weeks. The Global Physical Activity Questionnaire was used to monitor the physical activity of the participants at each visit. At baseline and 12 weeks, blood samples were drawn after ≥12 h of fasting. Fasting plasma glucose (FPG) level was analyzed using the hexokinase method. Plasma insulin concentration was measured using a radioimmunoassay (Linco, St Louis, MO, USA). These two variables were used to calculate the homoeostatic model assessment for insulin resistance (HOMA-IR) as HOMA-IR = insulin (μI U/ml) × FPG (mg/dl)/22.5. Total cholesterol, high-density lipoprotein-cholesterol (HDL-C), and low-density lipoprotein-cholesterol (LDL-C) levels were measured using homogeneous enzymatic assays, and levels of triglycerides (TG) were measured using a glycerol-3-phosphate oxidase peroxide method. Aspartate and alanine aminotransferase (using the NADH-UV method), blood urea nitrogen (urease/glutamate dehydrogenase method), and creatinine (Jaffe’s kinetic method) were measured at the central laboratory of each medical center. Estimated glomerular filtration rate was calculated using the creatinine-based Chronic Kidney Disease Epidemiology Collaboration equation. Urine and hematological parameters, including white blood cells, red blood cells, hemoglobin, hematocrit, platelets, mean red blood cell volume, and mean red blood cell hemoglobin of participants, were analyzed for safety. Systolic and diastolic blood pressure and pulse rate were measured on the left arm after a resting period using an automatic blood pressure monitor (Omron Healthcare Co. Ltd, Kyoto, Japan) in the sitting position twice on the same day. At every visit, the participants were kept in a stable state for at least 10 min, and then pulse and blood pressure were measured using the same equipment by the same researcher. Adverse events were monitored at 6 and 12 weeks.

### 2.3. Outcome measurements

The primary outcome was the change in body fat mass at baseline and 12 weeks, determined using dual-energy X-ray absorptiometry (DEXA, Lunar Prodigy Advance; GE, Wisconsin, USA). DEXA, a high-precision X-ray technique used to estimate bone mineral density with a short scanning time, measures fat mass and bone-free lean mass of the participants in the supine position. The secondary outcomes included body composition (lean body mass and body fat percentage) measured using DEXA; the abdominal adipose tissue areas (total abdominal fat areas, visceral fat area, and subcutaneous fat area) were obtained via computed tomography (CT) scans (SOMATOM Definition Flash; Siemens AG, Erlangen, Germany). A 10 mm CT slice scan was acquired at the umbilical level to measure subcutaneous and visceral fat areas by measuring the mean value of all pixels within the range of –190 to –30 Hounsfield unit at two medical centers. Changes in body weight, waist circumference, and hip circumference; lipid profile; and blood glucose levels at the baseline and 12 weeks were analyzed. Body weight was measured using a digital scale to the nearest 0.1 kg wearing lightweight clothes. Height was measured barefoot to the nearest 0.1 cm using an electronic scale. Waist and hip circumferences were measured using a plastic measuring tape to the nearest 0.1 cm.

### 2.4. Statistical analysis

The study sample size was calculated by accounting for a type II error, 95% confidence interval, α = 0.05, β = 0.2, and statistical power of 80% between the intervention groups vs. control based on a previous study (29). Assuming that the difference (mean ± SD) between the groups for the changes in body fat mass after ingestion of the test food was −2.51 ± 4.00 kg, the number of subjects in each group was estimated as 40. Therefore, considering a drop-out rate of 20%, 100 participants (50 persons per group) were enrolled. Comparisons of baseline characteristics were analyzed using Student’s *t*-test for continuous variables and the chi-square test for categorical variables. The results of continuous variables are expressed as mean ± standard error (SE), and categorical variables are expressed as the number of subjects. Adherence to the study product between the two groups was assessed using Student’s *t*-test, and all values are presented as mean ± SE. An intention-to-treat analysis was performed to evaluate outcomes. A linear mixed-effect model with random and fixed effects was applied to estimate the between-group or within-group changes for continuous variables, including outcomes adjusted for the institute, safety parameters, daily dietary intake, and physical activity. The models tested the effect of the group, time (week), and group × time (week) interaction. The results of the linear mixed-effects model are expressed as the least squares (LS) mean ± SE. Fisher’s exact test was used to compare the differences between the groups for categorical variables in urine tests and adverse events. McNemar’s test was used to compare parameters within each group between the baseline and 12-week follow-up. Statistical analysis was performed using SAS version 9.4 (SAS Institute, Cary, NC, USA), and a *p*-value < 0.05 was considered statistically significant, and missing values were not imputed.

## 3. Results

### 3.1. Baseline characteristics and participant flow

Ninety-five participants completed all visits, however, two participants with <80% product compliance were excluded from the data analysis (Figure 1). Consequently, 93 individuals were included in the safety analysis per protocol, with more than 95% adherence. No significant adverse events were observed during the study period. The baseline characteristics of the study participants are shown in Table 1. There were no significant differences in baseline characteristics between the two groups.

**TABLE 1:** Baseline characteristics of participants^a^.

Variables	Curezyme–LAC (*N* = 50)	Placebo (*N* = 50)	*p*-value^b^
Male sex, *n* (%)	14 (36.0)	19 (38.0)	0.288
Age (years)	50.8 ± 1.0	49.1 ± 0.9	0.197
Body weight (kg)	74.5 ± 1.6	77.5 ± 1.3	0.144
Body mass index (kg/m^2^)	28.2 ± 0.3	28.1 ± 0.3	0.746
Waist circumference (cm)	94.2 ± 0.9	95.6 ± 0.8	0.249
Hip circumference (cm)	103.4 ± 0.6	103.9 ± 0.7	0.607
Visceral adipose tissue area (cm^2^)	270.4 ± 9.9	261.7 ± 10.3	0.547
Subcutaneous adipose tissue area (cm^2^)	144.1 ± 5.4	154.5 ± 6.9	0.242
Fat free mass (kg)	45.4 ± 1.3	48.0 ± 1.2	0.151
Alcohol drinker, *n* (%)	24 (48.0)	29 (58.0)	0.316
Alcohol amount (SD/week)	5.1 ± 2.5	4.4 ± 1.1	0.804
Smoker, *n* (%)	10 (20.0)	14 (28.0)	0.349
Smoking amount (cigarettes/day)	1.6 ± 0.6	3.0 ± 0.8	0.177
Energy intake (kcal/day)	1489.1 ± 66.2	1638.2 ± 64.0	0.109
Physical activity (MET-min/week)	1166.0 ± 135.1	1220.4 ± 131.8	0.774

^a^All such values are presented as Mean ± SE (all such values), SD, standard drink; MET, metabolic equivalent task.

^b^Student’s t-test for continuous variables and the chi-square test for categorical variables were used to compare the difference between the groups.

### 3.2. Efficacy outcomes

**FIGURE 2:**
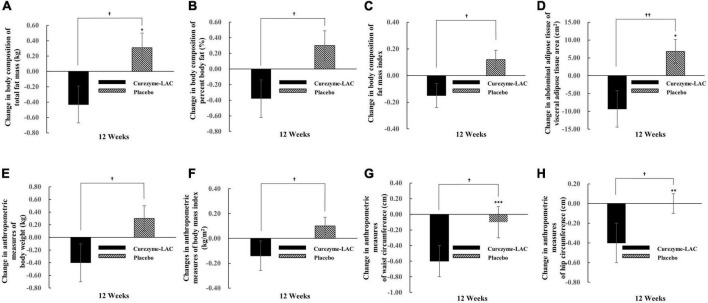
Changes in body composition, abdominal adipose tissue and anthropometric measurements in individuals receiving Curezyme-LAC group or placebo group. **(A)** Changes in body composition of total fat mass (kg), **(B)** changes in body composition of percent body fat (%), **(C)** changes in body composition of fat mass index, **(D)** changes in abdominal adipose tissue of visceral adipose tissue area (cm^2^), **(E)** changes in anthropometric measures of Body weight (kg), **(F)** changes in anthropometric measures of body mass index (kg/m^2^), **(G)** changes in anthropometric measures of waist circumference (cm), **(H)** changes in anthropometric measures of hip circumference (cm). Data are expressed as the mean ± SE. Linear mixed-effect model adjusted with institute and MEDFICTS for 12 weeks was used to analyze the group x period (week) for 12 weeks. *Indicates a significant difference from the baseline (*p* < 0.05; repeated measures ANOVA Contrast test). **Indicates a significant difference from the baseline (*p* < 0.01; repeated measures ANOVA Contrast test). ***Indicates a significant difference from the baseline (*p* < 0.001; repeated measures ANOVA Contrast test).^†^Indicates significant differences between the Curezyme-LAC group and the placebo group (*p* < 0.05; repeated measures ANOVA Contrast test), ^†⁣†^indicate significant differences between the Curezyme-LAC group and the placebo group (*p* < 0.01; repeated measures ANOVA Contrast test).

For the within-group comparisons, the changes in body composition parameters between baseline (adjusted to zero) and week 12 follow-up are shown in Figure 2. There were statistically significant LS mean changes in total fat mass (−0.43 ± 0.24 vs. 0.31 ± 0.19 g; *p* = 0.011, Figure 2A), percent body fat (−0.38 ± 0.22 vs. 0.30 ± 0.23 g; *p* = 0.026, Figure 2B), fat mass index (−0.15 ± 0.09 vs. 0.12 ± 0.07; *p* = 0.013, Figure 2C) in the Curezyme–LAC group compared with the placebo group. A significantly greater reduction in visceral adipose tissue (VAT) was observed in the Curezyme–LAC group after 12 weeks (−9.3 ± 5.1 vs. 6.8 ± 3.4 cm^2^; *p* = 0.008, Figure 2D). The anthropometric variables of body weight (−0.4 ± 0.3 vs. 0.3 ± 0.2 kg; *p* = 0.021, Figure 2E), BMI (−0.14 ± 0.12 vs. 0.10 ± 0.07 kg/m^2^); *p = 0.028*, Figure 2F), waist circumference (−0.6 ± 0.2 vs. −0.1 ± 0.2 cm; *p* = 0.018, Figure 2G) and hip circumference (−0.4 ± 0.2 vs. 0.0 ± 0.1 cm; *p = 0.019*) were decreased in the Curezyme–LAC group compared to the placebo group, whereas the waist-to-hip ratio did not show significant changes.

Body composition, abdominal adipose tissue, and anthropometric parameters are shown in Table 2. The values of fat mass in body composition were significantly improved in the Curezyme–LAC group compared with those in the placebo group at 12 weeks (29.2 ± 0.6 vs. 28.5 ± 0.6 kg, *p* = 0.042), whereas in the placebo group, none of the body composition parameters were significantly improved from baseline values. In comparisons between groups, the values of total fat mass, percent body fat, and fat mass index were significantly improved in the Curezyme–LAC group compared to the placebo group at 12 weeks (*p* = 0.011, *p* = 0.026, and *p* = 0.013, respectively) (Table 2). The values of VAT area in abdominal adipose tissue parameters significantly improved in the Curezyme–LAC group compared to the placebo group at 12 weeks (160.3 ± 6.2 vs. 135.2 ± 6.3 cm, *p = 0.028*), whereas in the placebo group, none of the abdominal adipose tissue parameters significantly improved from baseline values during the study period. In comparison between groups, the values of VAT were significantly improved in the Curezyme–LAC group compared with those in the placebo group at 12 weeks (*p* = 0.008). The values of body weight, waist circumference, and hip circumference in anthropometric parameters significantly improved in the Curezyme–LAC group compared to the placebo group at 12 weeks (77.8 ± 1.4 vs. 74.1 ± 1.4 kg, *p* = 0.044; 95.5 ± 0.9 vs. 93.6 ± 0.9 cm, *p* < 0.001; 103.9 ± 0.7 vs. 103.0 ± 0.7 cm, *p* = 0.003), whereas in the placebo group, none of the anthropometric parameters significantly improved from baseline values during the study period. In comparison between groups, body weight, BMI, waist circumference, and hip circumference significantly improved in the Curezyme–LAC group compared to the placebo group at 12 weeks (*p* = 0.021, *p* = 0.028, *p* = 0.018, and *p* = 0.019, respectively). No significant differences were observed in the blood lipid profiles and glucose metabolism between the study groups (Supplementary Table 3).

**TABLE 2:** Body composition, abdominal adipose tissue, and anthropometric measurements^a^.

Variables	Intention-to-treat population						
	Curezyme–LAC group (*n* = 50)			Placebo group (*n* = 50)			Placebo/ Curezyme–LAC
	Baseline	12 weeks	*p*-value^b^	Baseline	12 weeks	*p*-value^b^	*p*-value^c^
**Body composition**							
Total fat mass, kg	28.90 ± 0.63	28.46 ± 0.63	0.042	28.78 ± 0.64	29.16 ± 0.64	0.113	0.011
Percent body fat, %	39.29 ± 0.79	38.90 ± 0.79	0.080	37.81 ± 0.80	38.13 ± 0.79	0.152	0.026
Fat mass index	11.06 ± 0.27	10.90 ± 0.27	0.052	10.56 ± 0.27	10.71 ± 0.27	0.106	0.013
Lean mass, kg	42.94 ± 1.21	42.97 ± 1.20	0.828	45.39 ± 1.22	45.36 ± 1.21	0.565	0.575
Fat free mass, kg	45.30 ± 1.26	45.32 ± 1.25	0.860	47.76 ± 1.27	47.74 ± 1.26	0.605	0.624
Lean mass index	16.19 ± 0.25	16.21 ± 0.25	0.818	16.43 ± 0.25	16.42 ± 0.25	0.527	0.542
**Abdominal adipose tissue**							
Total adipose tissue area, cm^2^	414.49 ± 12.55	405.81 ± 12.68	0.227	416.17 ± 12.58	423.2 ± 12.6	0.336	0.126
Visceral adipose tissue area, cm^2^	143.80 ± 6.20	135.25 ± 6.27	0.028	154.95 ± 6.22	160.3 ± 6.2	0.108	0.008
Subcutaneous adipose tissue area, cm^2^	270.62 ± 10.48	270.55 ± 10.57	0.953	261.33 ± 10.50	262.66 ± 10.53	0.879	0.881
**Anthropometric measures by** **DEXA**							
Body weight, kg	74.55 ± 1.44	74.09 ± 1.44	0.044	77.46 ± 1.44	77.81 ± 1.44	0.205	0.021
Body mass index, kg/m^2^	28.25 ± 0.30	28.11 ± 0.30	0.076	28.07 ± 0.30	28.20 ± 0.30	0.180	0.028
Waist circumference, cm	94.24 ± 0.85	93.60 ± 0.85	<0.001	95.60 ± 0.85	95.55 ± 0.85	0.647	0.018
Hip circumference, cm	103.44 ± 0.67	103.04 ± 0.67	0.003	103.86 ± 0.67	103.94 ± 0.67	0.744	0.019
Waist-to-hip ratio	0.91 ± 0.01	0.91 ± 0.01	0.098	0.92 ± 0.01	0.92 ± 0.01	0.403	0.560

^a^All such values are presented as LS mean ± SE.

^b^P-value for group*time effect. Linear mixed-effect model adjusted to compare the changes for 12 weeks within each group.

^c^Linear mixed-effect model adjusted to analyze the difference between groups.

### 3.3. Daily dietary intake, physical activity, and safety assessment

No significant changes were noted in the daily dietary intake and physical activity between the two groups after the 12-week intervention (Supplementary Table 4). There were no serious adverse reactions requiring any participant to discontinue the use of Curezyme–LAC or hospitalization with regards to the occurrence, type, degree of symptoms, and relevance to intervention (Supplementary Table 5). The symptom severities of adverse reactions were mild, and any relationship with the intervention was not considered relevant. There were no deaths or serious adverse events requiring hospitalization. In addition, there were no clinically significant abnormal measurements of vital signs in physical examinations, hematological parameters, and blood chemistry parameters in either group (Supplementary Table 6).

## 4. Discussion

To the best of our knowledge, this is the first study to demonstrate that grains fermented by *B. coagulans* (Curezyme–LAC) reduce visceral fat mass. Curezyme–LAC supplementation significantly decreased visceral fat area (Figure 2) without any change in dietary intake or physical activity and decreased fat mass, body weight, and waist circumference (Figure 2 and Table 2, Supplementary Table 4). Several studies on people with obesity have attempted to lower the risk of cardiovascular disease by reducing visceral fat using various foods, such as fermented grains, using microbiota, other than anti-obesity drugs. A previous clinical trial showed that *Lactobacillus plantarum*-fermented barley extract intervention reduced the fat mass, percentage of fat, and visceral fat grade measured by a body composition analyzer and the fasting blood glucose, HbA1c, and TG levels in participants with metabolic syndrome (30). However, our study differs from previous studies in that it was the first to prove that applying grains fermented by *B. coagulans* in humans reduces visceral fat areas measured by CT [gold-standard measurements of visceral fat (31)] and improves several anthropometric indices such as weight loss and waist circumference.

In a preliminary study with obese mice induced by a high-fat diet, fermented mixed grain supplementation significantly decreased body weight, body fat mass, and plasma lipid levels in a dose-dependent manner, with an improvement in impaired glucose metabolism, hepatic steatosis, and inflammatory cytokines (14). Several studies have supported the use of fermented grains and their anti-obesity effects via an increase in β-oxidation and a decrease in fat synthesis (32, 33). Fasting blood glucose levels significantly decreased with improved glucose tolerance in the high-dose fermented mixed grain group. Furthermore, an animal study revealed that treatment with high-dose fermented mixed grains is efficacious in altering plasma adipokine and inflammatory cytokine concentrations (34). Curezyme–LAC supplementation might have the potential to ameliorate the risk of developing obesity-related comorbidities induced by the secretion of pro-inflammatory cytokines, although the causes for increasing visceral adipose vary in different individuals (35). Supporting our findings, Xiao et al. reported that fermented barley extracts enriched with total protein and polyphenol compounds significantly reduced body weight, abdominal adipose tissue weight, epididymal adipose tissue weight, and serum lipid levels in male rats (30). In another crossover study, the consumption of soybeans fermented by *B. natto* in overweight individuals with impaired glucose tolerance improved insulin sensitivity and oxidative stress and significantly reduced total and LDL-C levels (36). Akamine et al. studied Okinawans with metabolic syndrome in their study. Fermented brown rice beverages increase the abundance of bacteria capable of producing butyrate, lactate, and ethanol, particularly *Sutterella*, which lower blood glucose levels (37).

The pathophysiology of obesity is multifactorial, and the role of intestinal microflora in various causes has recently attracted attention. In both mice and humans with obesity, Gram-negative Bacteroidetes and Gram-positive Firmicutes are the dominant species in the gut (38, 39). In contrast, preclinical evidence supporting the “anti-obesity” effect of probiotics comes primarily from studies of probiotics belonging to the genus *Lactobacillus* and strains of *Bifidobacterium* (40, 41). They are the most commonly used bacteria in probiotic foods (42) as potential probiotics with anti-obesity effects, but they are not resistant to heat treatment (43, 44). *B. coagulans* is a spore-forming bacterium resistant to high temperatures, has probiotic activity, and could be used to develop functional foods. Although a few studies have shown the effects of *B. coagulans* on the microbiome, several beneficial effects have been reported. Several studies have demonstrated various health-promoting effects of supplementation with *B. coagulans* spores, including regulating gastrointestinal disorders, stimulating the immune system, and lowering cholesterol (45–47). As facultative anaerobic bacteria, *B. coagulans* strains can help create an anaerobic and acidic intestinal environment, thereby promoting the growth of probiotics. It has proven beneficial for the growth of anaerobic microorganisms such as *Lactobacillus* and *Bifidobacterium* (48). The ability of *B. coagulans* to improve the anti-obesity effect of these microbes by increasing the intestinal environment in which *Lactobacillus* or *Bifidobacterium* could survive is likely to explain the findings of this study.

Several studies have shown that visceral fat is more associated with cardiovascular risk factors than subcutaneous fat (49). The results of the current study showed that Curezyme–LAC could be a beneficial food ingredient to reduce the accumulation of VAT, as well as indicators relevant to obesity, such as body fat mass, weight, and waist circumference in obese individuals. In addition, it may contribute to reducing various cardiometabolic risks owing to decreased visceral fat.

One potentially ideal strategy for obesity treatment is to manipulate the gut microbiome. This treatment is safe because it has no reported serious side effects and may be suitable for long-term use (50). It can be an alternative treatment that can be further incorporated into existing obesity treatments. Therefore, this study is meaningful because it showed some potential in reducing fat mass among people with obesity with a product using the *B. coagulans* strain, which has not yet been studied as a probiotic treatment. In addition, there were no serious adverse reactions reported.

However, this study had some limitations. This study was conducted over a short period because it had a small number of participants. Moreover, this study was conducted in a single hospital, limiting its generalizability. Therefore, a large cohort study or meta-analysis of human participants is needed to confirm whether Curezyme–LAC has anti-obesity effects. Moreover, the mechanism of Curezyme–LAC involved in the anti-obesity effect is unclear. Since the thermal stability of *B. coagulans* within this product and the analysis of metabolites or bioactive compounds of the product have not been demonstrated, this study cannot suggest a mechanism for reducing visceral fat. Therefore, if research is conducted to uncover this mechanism in the future, it could be used as a potential treatment for obesity. In addition, future studies should clarify whether Curezyme–LAC affects glucose and fat metabolism and visceral fat through modulation of the gut microbiome, insulin resistance, or chronic inflammation in humans.

## 5. Conclusion

We demonstrated that grains fermented by *B. coagulans* could remarkably reduce visceral fat in individuals with obesity. Furthermore, our results showed that grains fermented by *B. coagulans* are capable of lowering the total fat mass, body weight, and waist circumference without changes in dietary intake and physical activity in participants with obesity. In addition, it has the potential as a functional health food with proven anti-obesity effects that can be used for a long time without serious side effects. Further large-scale studies with representative populations are needed to confirm these findings.

## Data availability statement

The raw data supporting the conclusions of this article will be made available by the authors, without undue reservation.

## Ethics statement

The studies involving human participants were reviewed and approved by the Institutional Review Board (IRB) of Seoul National University Hospital as well as by Seoul National University Bundang Hospital and adhered to the Declaration of Helsinki (IRB No. H-2002-098-1103). The patients/participants provided their written informed consent to participate in this study.

## Author contributions

EC, JK, and BC conceived the study and executed the trial. EC, JSL, JK, BC, and YCY reviewed the study protocol. JSL and YCY performed the statistical analysis. EC, JSL, JK, BC, YCY, YCS, HK, SG, and SK further assisted in the investigation and interpretation, critically reviewed the manuscript, and approved the final version. EC initiated the manuscript and JSL, JK, and BC further assisted. All authors contributed to the article and approved the submitted version.
