# Supplementary material for: Efficacy of fermented grain using Bacillus coagulans in reducing visceral fat among people with obesity: a randomized controlled trial
Source: Front Nutr. 2023 Apr 17;10:1148512. doi: 10.3389/fnut.2023.1148512 (PMC10149940; doi:10.3389/fnut.2023.1148512)
Supplement: Supplementary file 1 [file Table_1.DOCX]

Supplementary Material

Efficacy of Fermented Grain using *Bacillus coagulans* in Reducing Visceral Fat Among People with Obesity: A Randomized Controlled Trial

Eunbyul Cho*, Ju Young Kim^†^, Belong Cho^†^, Joong Su Lee, Yeo Cho Yoon, Yong Chul Shin, Hyerim Kim, Siye Gil, Sohye Kim

*** Correspondence:** Ju Young Kim: kkamburi@gmail.com

Belong Cho: belong@snu.ac.kr

†These corresponding authors contributed equally to this work.

**Supplementary Table 1. Inclusion and exclusion criteria for participant recruitment**

| Inclusion Criteria |
| --- |
| ∙ Healthy volunteers aged ≥ 40 and ≤ 65 years. |
| ∙ Body mass index (BMI) ≥ 25 and ≤ 35 kg/m^2^ |
| ∙ Waist circumference of ≥ 90 cm for males and ≥ 85 cm for females |
| ∙ Agree not to change their current diet or exercise program during the entire study period. |
| Exclusion Criteria |
| ∙ Current use of dietary supplements (including probiotics and prebiotics) or medications that could affect  body weight, lipid profile, glucose metabolism, immunity and inflammation, and gut health within 1 month  before the screening visit (appetite suppressants and stimulants, antidepressants, diuretics, β-blockers,  contraceptives, hormonal agents, lipid-lowering drugs, glucose-lowering agents, laxatives, anti-  inflammatory drugs, inflammatory bowel disease treatment). |
| ∙ Unstable hypertension (systolic blood pressure >160 mmHg or diastolic blood pressure > 100 mmHg). |
| ∙ Subjects who Diagnosed as diabetes mellitus or fasting blood sugar ≥ 126 mg/dL or HbA1c ≥ 6.5% |
| ∙ Subjects who have abnormal liver function (≥ three times the normal range of serum aspartate  aminotransferase or alanine aminotransferase levels) or currently being treated with hepatobiliary  diseases |
| ∙ Subjects who are stage 4 or stage 5 chronic kidney disease (estimated glomerular filtration rate,  eGFR ≤ 30 mL/min/1.73 m^2^) |
| ∙ Untreated and unstable hypothyroidism |
| ∙ History of Cushing syndrome |
| ∙ History of past and present severe neurological or psychological disorders (depression, schizophrenia,  epilepsy, alcohol addiction, drug addiction, and eating disorder) |
| ∙ History of cerebrovascular diseases (cerebral infarction and cerebral hemorrhage), heart disease (angina  pectoris, myocardial infarction, cardiac failure, and arrhythmia), malignant tumors |
| ∙ Chronic obstructive pulmonary disease, rheumatic diseases, or on-going medical treatment of  autoimmune disease |
| ∙ Severe musculoskeletal disorder which kept participants from doing regular exercise |
| ∙ Subjects with a commercial weight loss program or calorie-restricted dietary treatment in the  previous 3 months |
| ∙ Subjects with weight change > 5% within 3 months prior to the study |
| ∙ History of depression, schizophrenia, alcoholism, drug addiction, or mental illness. |
| ∙ Subjects who have done consistent high-intensity exercise for more than 3 months |
| ∙ Subjects who are pregnant, lactating, or planning pregnancy |
| ∙ History of allergies or hypersensitivity to any of study products or ingredients |

**Supplementary Table 2. Formulation of Curezyme–LAC group and placebo group preparations**

| Ingredients | Curezyme–LAC group | | Placebo group | |
| --- | --- | --- | --- | --- |
|  | Content (mg) | Content (%) | Content (mg) | Content (%) |
| Curezyme–LAC | 20,001 | 66.67 | 0 | 0.00 |
| Steamed mixed grains powder |  | 0.00 | 20,001 | 66.67 |
| Fruit concentrate mix | 4,899 | 16.33 | 4,899 | 16.33 |
| Sweetener | 300 | 1.00 | 300 | 1.00 |
| Excipients | 4,800 | 16.00 | 4,800 | 16.00 |
| Total | 30,000 | 100.00 | 50,000 | 100.00 |

**Supplementary Table 3. Blood lipid profiles and glucose metabolism^1^**

| **Variables** | **Curezyme–LAC group (n=50)** | | | ***P*-value^2^** | **Placebo group(n=50)** | | | ***P*-value^2^** | ***P*-value^3^** |
| --- | --- | --- | --- | --- | --- | --- | --- | --- | --- |
|  | **Baseline** | **12 weeks** | **Change** |  | **Baseline** | **12 weeks** | **Change** |  |  |
| **Blood lipid profiles** | | | | | | | | | |
| Total cholesterol (mg/dL) | 216.3 ± 5.1 | 215.9 ± 5.2 | -0.3 ± 4.3 | 0.942 | 216.4 ± 5.1 | 218.8 ± 5.2 | 2.9 ± 3.5 | 0.435 | 0.547 |
| Triglyceride (mg/dL) | 131.9 ± 10.0 | 124.7 ± 10.1 | -7.6 ± 8.0 | 0.342 | 142.0 ± 10.0 | 140.6 ± 10.0 | 1.0 ± 8.2 | 0.913 | 0.453 |
| HDL-cholesterol (mg/dL) | 54.8 ± 1.6 | 55.0 ± 1.6 | 1.2 ± 3.9 | 0.849 | 54.2 ± 1.6 | 55.1 ± 1.6 | 2.9 ± 2.8 | 0.458 | 0.697 |
| LDL-cholesterol (mg/dL) | 141.1 ± 4.3 | 142.2 ± 4.4 | 0.2 ± 1.1 | 0.727 | 141.7 ± 4.3 | 144.3 ± 4.4 | 0.9 ± 1.2 | 0.366 | 0.696 |
| VLDL-cholesterol (mg/dL) | 26.4 ± 2.0 | 24.9 ± 2.0 | -1.5 ± 1.6 | 0.342 | 144.3 ± 4.4 | 28.1 ± 2.0 | 0.2 ± 1.6 | 0.913 | 0.453 |
| **Glucose metabolism** | | | | | | | | | |
| Blood glucose (mg/dL) | 96.1 ± 1.4 | 96.4 ± 1.4 | 0.2 ± 1.3 | 0.902 | 95.3 ± 1.4 | 95.3 ± 1.4 | 0.4 ± 1.3 | 0.779 | 0.912 |
| Insulin (μIU/mL) | 9.2 ± 0.9 | 10.1 ± 0.9 | 0.9 ± 1.0 | 0.224 | 8.2 ± 0.9 | 8.2 ± 0.9 | 0.0 ± 0.4 | 0.973 | 0.377 |
| HOMA-IR | 2.2 ± 0.2 | 2.5 ± 0.2 | 0.4 ± 0.3 | 0.121 | 2.0 ± 0.2 | 2.0 ± 0.2 | 0.0 ± 0.1 | 0.983 | 0.265 |

^1^ All such values are presented as LS mean ± SE. HDL-C, high density lipoprotein cholesterol; LDL-C, low density lipoprotein cholesterol; VLDL-C, very low-density lipoprotein; HOMA-IR, homeostatic model assessment for insulin resistance.

^2^ Linear mixed-effect model adjusted with institute was used to analyze the difference within each group.

^3^ P-value for group*time effect. Linear mixed-effect model adjusted to compare the changes for 12 weeks between the groups.

**Supplementary Table 4. Dietary intake and physical activity^1^**

| **Variables** | **Curezyme–LAC group**  **(n = 50)** | ***P*-value^2^** | **Placebo group  (n = 50)** | ***P*-value^2^** | ***P*-value^3^** | | |
| --- | --- | --- | --- | --- | --- | --- | --- |
|  |  |  |  |  | **Group** | **Week** | **Group×Week** |
| **Dietary intake** | | | | | | | |
| Energy (kcal/d) | |  |  |  |  |  |  |
| Week 0 | 1489.1 ± 66.7 | 0.817 | 1638.2 ± 66.7 | 0.252 | 0.036 | 0.267 | 0.76 |
| Week 6 | 1524.1 ± 67.0 |  | 1718.8 ± 67.0 |  |  |  |  |
| Week 12 | 1500.9 ± 67.6 |  | 1696.7 ± 67.3 |  |  |  |  |
| Carbohydrate (g/d) | |  |  |  |  |  |  |
| Week 0 | 219.8 ± 9.8 | 0.587 | 233.8 ± 9.8 | 0.604 | 0.198 | 0.268 | 0.825 |
| Week 6 | 225.9 ± 9.8 |  | 245.8 ± 9.8 |  |  |  |  |
| Week 12 | 224.2 ± 9.9 |  | 237.9 ± 9.9 |  |  |  |  |
| Protein (g/d) | |  |  |  |  |  |  |
| Week 0 | 58.6 ± 2.9 | 0.921 | 63.7 ± 2.9 | 0.054 | 0.053 | 0.299 | 0.313 |
| Week 6 | 60.5 ± 2.9 |  | 66.5 ± 2.9 |  |  |  |  |
| Week 12 | 58.3 ± 3.0 |  | 68.5 ± 3.0 |  |  |  |  |
| Fat (g/d) |  |  |  |  |  |  |  |
| Week 0 | 41.1 ± 2.7 | 0.914 | 47.9 ± 2.9 | 0.314 | 0.012 | 0.606 | 0.784 |
| Week 6 | 41.7 ± 2.8 |  | 50.7 ± 2.8 |  |  |  |  |
| Week 12 | 41.4 ± 2.8 |  | 50.6 ± 2.8 |  |  |  |  |
| Sodium (mg/d) | |  |  |  |  |  |  |
| Week 0 | 3401.4 ± 228.0 | 0.332 | 3959.2 ± 228.0 | 0.055 | 0.013 | 0.123 | 0.657 |
| Week 6 | 3393.7 ± 229.4 |  | 4198.7 ± 229.4 |  |  |  |  |
| Week 12 | 3598.2 ± 231.9 |  | 4346.9 ± 230.6 |  |  |  |  |
| **Physical activity (MET-min/wk)** | | | | | | | |
| Week 0 | 1166.0 ± 137.9 | 0.773 | 1220.4 ± 137.9 | 0.933 | 0.714 | 0.709 | 0.834 |
| Week 6 | 1103.5 ± 138.4 |  | 1213.1 ± 138.4 |  |  |  |  |
| Week 12 | 1192.1 ± 139.3 |  | 1227.9 ± 138.8 |  |  |  |  |

^1^ All such values are presented as LS mean ± SE.

^2^ Linear mixed-effect model adjusted with institute was used to analyze the difference within each group.

^3^ *P*-value for group*time effect. Linear mixed-effect model adjusted to compare the changes for 12 weeks between the groups.

**Supplementary Table 5. Adverse events of the study participants^1^**

| **Variables** | **Intention to treat population** | | |
| --- | --- | --- | --- |
|  | **Curezyme–LAC group (n=50)** | **Placebo group (n=50)** | ***P-value^2^*** |
| Adverse event | 3/3 | 3/3 | 1.000 |
| Serious adverse event | 0/0 | 0/0 | - |
| Type |  |  |  |
| Cough | 0/0 | 1/1 | 1.000 |
| Low back pain | 0/0 | 1/1 | 1.000 |
| Vaginitis | 0/0 | 1/1 | 1.000 |
| Dyspepsia, Gastrointestinal Manifestations | 2/2 | 0/0 | 0.495 |
| Diarrhea | 1/1 | 0/0 | 1.000 |
| Grade of symptom |  |  |  |
| Mild | 3/3 | 3/3 | 1.000 |
| Moderate | 0/0 | 0/0 | - |
| Severe | 0/0 | 0/0 | - |
| Relevance to Curezyme–LAC |  |  |  |
| Definitely related | 0/0 | 0/0 | - |
| Probably related | 0/0 | 0/0 | - |
| Possible related | 0/0 | 0/0 | - |
| Probably not related | 2/2 | 2/2 | 1.000 |
| Definitely not related | 1/1 | 1/1 | 1.000 |
| Unknown | 0/0 | 0/0 | - |

^1^Number of subjects/number of cases. AE, adverse event; SAE, serious adverse event.

^2^Fisher’s exact test was used to compare the difference between the groups.

**Supplementary Table 6. Changes in blood pressure and biomarkers in the Curezyme–LAC and placebo groups after 12 weeks^1^**

| **Variables** | **Intention to treat population** | | | | | | |
| --- | --- | --- | --- | --- | --- | --- | --- |
|  | **Curezyme–LAC group (n=50)** | | | **Placebo group (n=50)** | | | **Placebo/Curezyme–LAC** |
|  | **Baseline** | **12 weeks** | ***P-value^2^*** | **Baseline** | **12 weeks** | ***P-value^2^*** | ***P-value^3^*** |
| **Vital signs** |  |  |  |  |  |  |  |
| SBP, mmHg | 125.8 ± 1.9 | 127.1 ± 1.9 | 0.318 | 127.2 ± 1.7 | 124.5 ± 1.6 | 0.060 | 0.125 |
| DBP, mmHg | 79.4 ± 1.6 | 78.3 ± 1.5 | 0.445 | 80.4 ± 1.4 | 79.1± 1.1 | 0.289 | 0.379 |
| Pulse rate, beats/min | 74.0 ± 1.2 | 73.6 ± 1.5 | 0.658 | 76.6 ± 1.3 | 79.1 ± 1.4 | 0.031 | 0.082 |
| **Hematological parameters** |  |  |  |  |  |  |  |
| WBC, 10^3^/µL | 6.1 ± 0.2 | 5.8 ± 0.2 | 0.092 | 6.0 ± 0.2 | 6.4 ± 0.2 | 0.005 | 0.002 |
| RBC, 10^6^/µL) | 4.6 ± 0.1 | 4.6 ± 0.1 | 0.797 | 4.7 ± 0.1 | 4.8 ± 0.1 | 0.007 | 0.080 |
| Hb, g/dL) | 13.9 ± 0.2 | 13.8 ± 0.2 | 0.776 | 14.2 ± 0.2 | 41.3 ± 0.2 | 0.115 | 0.190 |
| Hct, % | 41.7 ± 0.5 | 41.7 ± 0.5 | 0.961 | 42.6 ± 0.5 | 43.2 ± 0.5 | 0.010 | 0.061 |
| PLT, 10^3^/µL | 258.0 ± 7.7 | 258.7 ± 7.7 | 0.854 | 266.0 ± 7.7 | 271.5 ± 7.7 | 0.151 | 0.377 |
| MCV, fL | 91.4 ± 0.7 | 91.2 ± 0.7 | 0.568 | 90.3 ± 0.7 | 90.1 ± 0.7 | 0.444 | 0.894 |
| MCH, pg | 30.4 ± 0.3 | 30.3 ± 0.3 | 0.300 | 30.1 ± 0.3 | 29.8 ± 0.3 | 0.015 | 0.317 |
| **Blood chemistry parameters** |  |  |  |  |  |  |  |
| ALT, IU/L | 25.9 ± 1.9 | 28.4 ± 2.9 | 0.101 | 26.7 ± 1.9 | 26.3 ± 2.4 | 0.967 | 0.231 |
| AST, IU/L | 22.4 ± 0.9 | 23.9 ± 1.3 | 0.058 | 24.1 ± 1.4 | 24.3 ± 1.7 | 0.858 | 0.219 |
| GGT, IU/L | 27.0 ± 3.9 | 24.5 ± 2.1 | 0.385 | 31.4 ± 6.2 | 28.2 ± 3.4 | 0.520 | 0.285 |
| BUN, mg/dL | 13.5 ± 0.5 | 13.7 ± 0.6 | 0.871 | 13.6 ± 0.5 | 14.0 ± 0.5 | 0.333 | 0.570 |
| Creatinine, mg/dL | 0.8 ± 0.0 | 0.8 ± 0.0 | 0.437 | 0.8 ± 0.0 | 0.8 ± 0.0 | 0.398 | 0.252 |
| Albumin, g/dL | 4.5 ± 0.0 | 4.5 ± 0.0 | 0.422 | 4.5 ± 0.0 | 4.5 ± 0.0 | 0.017 | 0.025 |
| eGFR, mL/min/1.73 m^2^ | 93.5 ± 1.8 | 94.6 ± 1.8 | 0.269 | 94.1 ± 1.8 | 93.5 ± 1.8 | 0.544 | 0.226 |

^1^LS mean ± SE (all such values). SBP, systolic blood pressure; DBP, diastolic blood pressure; WBC, white blood cell; RBC, red blood cell; Hb, hemoglobin; Hct, hematocrit; PLT, platelet; MCV, mean corpuscular volume; MCH, mean corpuscular hemoglobin; MCHC, mean corpuscular hemoglobin concentration; ALT, alanine aminotransferase; AST, aspartate aminotransferase; GGT, gamma-glutamyl transferase; BUN, blood urea nitrogen; eGFR, estimated glomerular filtration rate;

^2^P-value for group × period (week) effect. Linear mixed-effect model adjusted and MEDFICTS (Meats, Eggs, Frying Foods, In baked goods, Convenience foods, Table fats, Snack) for 12 weeks was used to compare the changes for 12 weeks between the groups.

^3^Linear mixed-effect model adjusted with institute and MEDFICTS for 12 weeks was used to analyze the difference within each group.
